# Virus-Inspired Design Principles of Nanoparticle-Based Bioagents

**DOI:** 10.1371/journal.pone.0013495

**Published:** 2010-10-19

**Authors:** Hongyan Yuan, Changjin Huang, Sulin Zhang

**Affiliations:** Department of Engineering Science and Mechanics, The Pennsylvania State University, University Park, Pennsylvania, United States of America; University of Florida, United States of America

## Abstract

The highly effectiveness and robustness of receptor-mediated viral invasion of living cells shed lights on the biomimetic design of nanoparticle(NP)-based therapeutics. Through thermodynamic analysis, we elucidate that the mechanisms governing both the endocytic time of a single NP and the cellular uptake can be unified into a general energy-balance framework of NP-membrane adhesion and membrane deformation. Yet the NP-membrane adhesion strength is a globally variable quantity that effectively regulates the NP uptake rate. Our analysis shows that the uptake rate interrelatedly depends on the particle size and ligand density, in contrast to the widely reported size effect. Our model predicts that the optimal radius of NPs for maximal uptake rate falls in the range of 25–30 nm, and optimally several tens of ligands should be coated onto NPs. These findings are supported by both recent experiments and typical viral structures, and serve as fundamental principles for the rational design of NP-based nanomedicine.

## Introduction

Viruses invade living cells via protein-mediated endocytosis [1], [2] or membrane fusion [3]. In the former case, the proteins (known as ligands) on the surface of viruses bind specifically with the complementary proteins (known as receptors) on the cell membrane. The ligand-receptor binding triggers a complex succession of biomechanical and biochemical events: docking, membrane wrapping, pinching off, and intracellular trafficking, etc. For example, a hepatitis C virus (HCV) [4], about 50 nm in size, is constituted of an inner core of RNA genetic materials, an icosahedral protective shell of protein, and a lipid envelope. HCVs infect specifically liver cells by endocytosis through the glycoproteins (ligands) on their lipid envelope. Once endocytosed, HCVs can be replicated in liver cells and bud off, continue to invade other liver cells, and subsequently cause liver cancer.

The highly effective and robust adhesion-driven process has raised many fundamental questions with regard to the physical principles harnessed by the evolutionary design of viruses. While it has long been known from biochemistry that the molecular recognition of receptors and ligands allows viral invasion to be type specific, it was only recently fully understood from mechanics point of view that viral invasion is also size selective [5], [6], [7], [8], [9], [10], [11], [12], [13], [14], [15], [16], [17]. Questions remain to be elucidated as to whether there exists an optimal ligand density for maximal uptake rate. Further, considering the robustness of material design principles exploited by nature via evolution, the effects of particle size and ligand density are likely interrelated. A thorough understanding of these fundamental issues is not only scientifically interesting, but also sheds light on the biomimetic design of nanoparticle (NP)-based therapeutics.

From a fundamental mechanics point of view, adhesion and membrane deformation play the roles of driving force and resistance to NP endocytosis, respectively. A rational biomimetic design of NPs should either reduce the resistance or enhance the adhesion to facilitate NP internalization. Indeed, it has both experimentally [10], [12], [13], [14], [18] and theoretically [5], [6], [16], [19], [20], [21] demonstrated that tailoring the size and shape of NPs alters the deformation resistance to curve the membrane, which explains the strong size and shape dependence of NP uptake properties. Yet few experimental studies have been attempted to tailor adhesion between NPs and cell membrane, despite that such modification could be accomplished by controlling the density of ligands coated onto the NP surface. In existing theoretical models [6], [22] ligand density is rarely treated as a design parameter despite its significant role indicated from viral infection processes.

In this article, we aim to establish guiding principles for the biomimetic design of NPs with high uptake rate, one of the key parameters that assess the efficacy of NP-based therapeutics. Noting that correlating the biophysical parameters of NPs with the uptake rate may analytically be complex, we circumvent the difficulty by separately deriving the endocytic time of a single NP and the equilibrium cellular uptake when immersing the cell in a solution with dispersed NPs. The endocytic time and cellular uptake together indicate the uptake rate. From thermodynamic analyses, we reveal that particle size and ligand density interrelatedly govern the uptake rate. The interrelated effects can be interpreted from a general framework of energy balance between NP-membrane adhesion and membrane deformation. The interrelation suggests that tailoring only one design parameter may not be effective to achieve high uptake rate. We construct a phase diagram of the uptake rate in the space of particle size and ligand density, which may serve as a design map for NP-based therapeutics. Finally, we extend our discussions by including the effects of other relevant biophysical parameters.

## Results

### 1. General energetics of endocytosis

From an energetics point of view, NP engulfment by cell membrane is driven by adhesion but involves significant membrane deformation cost [23], where adhesion energy may stem from both non-specific and specific interactions [24]. For a general consideration, the adhesion energy density (per unit area) is denoted by 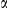. Since the NPs considered here are much smaller than the cell, it is reasonable to assume that cell membrane is locally flat at the NP-membrane adhering site and the effect of spontaneous curvature of cell membrane may be neglected. One then follows that the bending energy density is 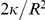, where *R* is the NP radius and 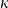 is the bending rigidity. Fully wrapping an NP involves a bending energy of 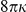, independent of particle size. A local energy balance between adhesion and bending yields a minimal particle radius 
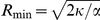
 below which adhesion energy is insufficient to drive wrapping.

Wrapping also involves pulling excess membrane area toward the wrapping site, for which work needs to be done to overcome membrane tension, denoted here by 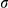. We define the degree of wrapping, denoted by 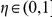, as the area fraction of wrapped NP surface. The NPs may associate with the membrane via three possible states: completely naked (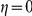), fully wrapped (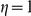), and partially wrapped (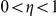). The membrane segment wrapped onto the NP stores both bending and stretching energies, denoted by 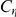 and 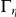, respectively. The curved membrane detaching from the contact to the NP contributes additional deformation energy 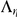, which has been previously derived with great clarity [23]. One notes 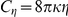 is linear, 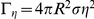 is quadratic, and 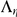 is nonlinear with respect to 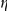 but vanishes when 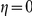 or 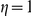. The total membrane deformation energy at the degree of wrapping 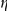 is written as 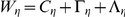.

Because of the nonlinearity of 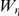, fully wrapping an NP needs to overcome an energy barrier [23]. However, as far as thermodynamics is concerned, a closed form of the total deformation energy at the fully wrapped state exists: 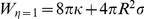. Equalizing the total deformation energy to the adhesion energy 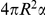 gives rise to a minimal particle radius, as
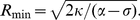
(1)The above equation indicates that there exists a critical adhesion strength 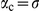 at which 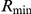 becomes infinite. This critical condition corresponds to the case that the adhesion is even insufficient to overcome membrane tension.

The total deformation energy at the fully wrapped state indicates a characteristic particle radius 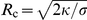 that weighs the relative significance of bending and membrane tension [23]. For 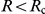, bending dominates the physics, while for 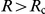 membrane tension dominates. One also notes that the energy of the fully wrapped state differs from that of the endocytosed state by a constant 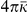 due to the topological change, where 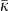 is the Gaussian bending rigidity. Since this energy trivially affects the final stage of wrapping, we ignore its effect in the following discussions.

### 2. Unique features of receptor-mediated endocytosis

Several unique features arise when endocytosis is receptor-mediated. First, as adhesion is supplied by ligand-receptor binding, wrapping of NPs requires diffusing receptors to the binding sites, thereby setting a characteristic time scale of endocytosis and limiting the uptake rate [6], [16]. Second, much like cleavage fracture (or crack healing) in crystals that involves discrete bond breaking (or formation) [25], [26], [27], NP wrapping through ligand-receptor binding proceeds in a discrete manner. The wrapping area in each discrete wrapping step can thus be tailored by the spacing of the ligands (i.e., the ligand density) coated on the NP surface. Finally, receptors, in addition to providing adhesion, also carry translational entropy [28], [29], [30]. This dual character renders the adhesion strength in receptor-mediated endocytosis a globally variable quantity, in distinct contrast to the adhesion strength between two inanimate objects that is commonly regarded as a material constant.

To reflect the discreteness of receptor-mediated endocytosis, it is convenient to set the cross-sectional area of the receptor as the unit area, denoted by 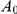, and 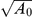 as the unit length. For typical transmembrane receptors, 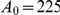 nm^2^. We assume that the receptors are initially uniformly distributed on the cell membrane with an initial receptor density 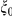. Given a spherical particle of radius *R* and coated with ligands of surface density 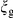, the maximum number of receptors accessible to the NP surface is 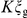, where 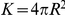. The binding energy of each ligand-receptor pair is denoted by 
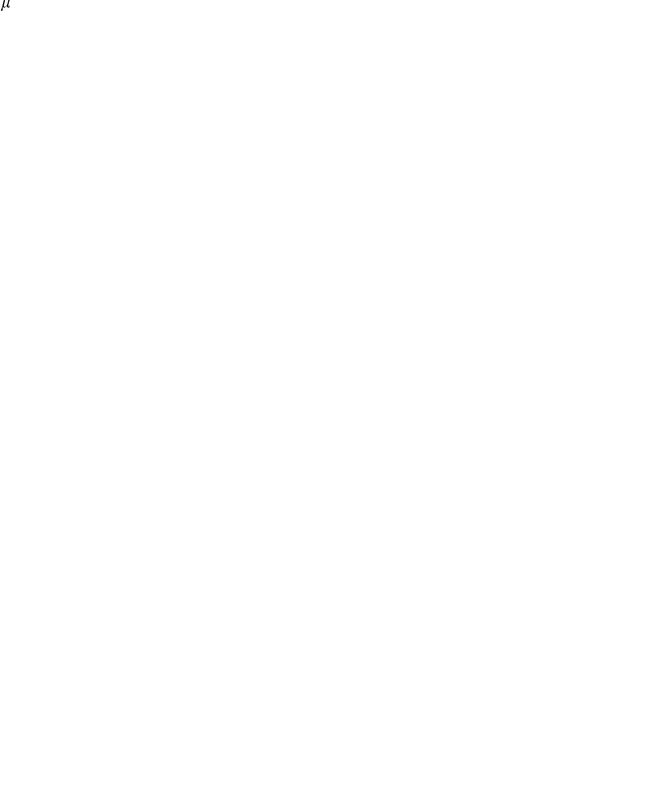
. The degree of wrapping can then be written as 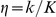, where 
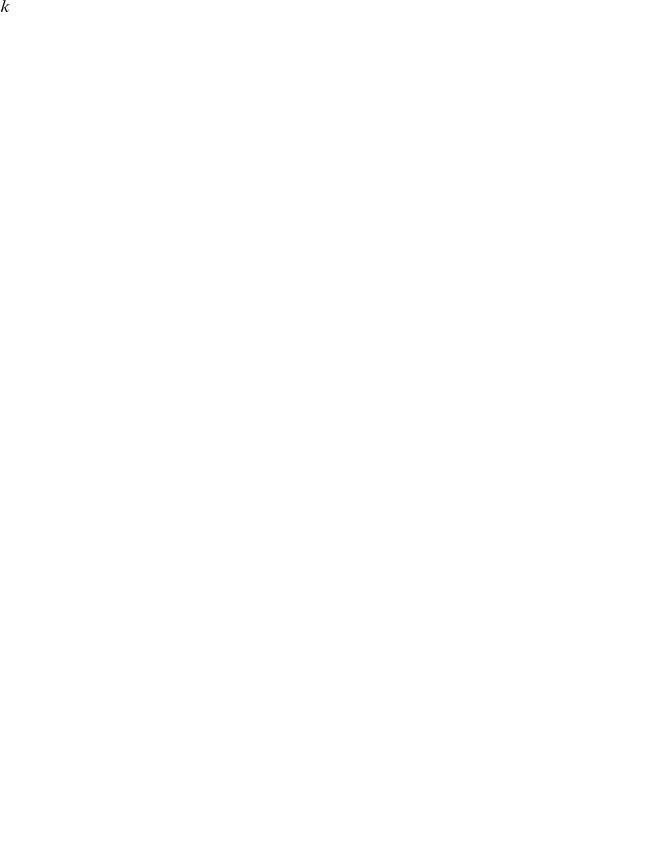
 is the wrapping area. The deformation energy density of the membrane in wrapping is 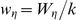. From hereafter, the functional dependence on 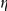 and *k* may be described interchangeably at given *K*.

The dual character of receptors suggests that the adhesion strength in receptor-mediated endocytosis can be decomposed into two components, i.e., 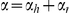, where 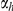 and 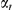 are the enthalpic and entropic components of the adhesion strength, respectively. The enthalpic component is simply 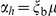, provided that the density of receptors bound onto the NP 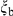 is known. Previous studies always assume that 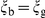 in order to simplify the analysis despite that 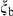 is significantly less than 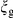 under certain conditions [7], [30]. Noting that the entropic adhesion strength is always negative and 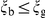, the adhesion strength reaches its maximum 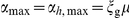 at 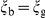. Under this extreme condition, one arrives at a minimal particle radius
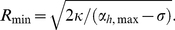
(2)A comparison between Eqs. (1) and (2) clarifies that Eq. (2) was derived from a “*local*” energy balance. Such a local consideration ignores the entropic effect of receptors, which represents the “*global*” aspects of adhesion. Equation (2) also manifests the interrelated effects of particle size and ligand density on NP endocytosis.

### 3. Endocytosis driven by variable adhesion strength

To measure the cellular uptake rate, experiments *in vitro* involve immersing biological cells into a solution with dispersed NPs [10], [13], [18]. It has been observed that the cellular uptake increases monotonically at the beginning and gradually reaches a plateau within several hours, indicating the obtainment of thermodynamic equilibrium [13], [14]. In this process, the diffusive receptors frequently change their binding targets (NPs) [20], [31]. The dynamic binding and debonding processes make direct analytical account of the time-varying cellular uptake (the uptake rate) intractable. Thus, our analysis involves solving the two sub-problems: the endocytic time of a single NP and the equilibrium cellular uptake when immersing the cell in a solution with dispersed NPs, both within a thermodynamic framework. The endocytic time and the cellular uptake together define a phenomenological uptake rate. Throughout our analysis, we highlight the global aspect of the adhesion strength originated from the entropy of diffusive receptors.

Unless otherwise mentioned, the following parameter values are used for our analysis: 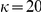, 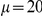, 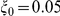, 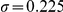, 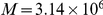, and 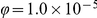. All the values are in the reduced units and physiologically relevant.

#### 3.1 Endocytic time of a single NP

The fact that wrapping necessitates receptors to diffuse from the far field to the wrapping site sets a diffusion-limited endocytic time. The endocytic time can thus be determined by formulating a diffusion problem that involves tracking the wrapping front of the cell membrane [16]. An alternative approach is to determine the total number of receptors required to fully wrap the NP [5], from which the endocytic time can be estimated. The latter approach is adopted in our following formulation.

**Figure 1:**
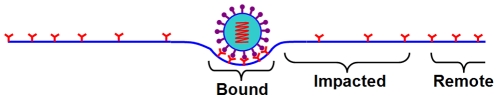
Schematics of endocytosis of a single NP. The membrane is partitioned into three regions due to the wrapping: the bound region of area 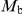, the impacted region of area 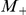, and the remote region of area 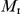.

Similar to crack extension or healing in a crystal lattice [25], [26], [27], the discrete wrapping of an NP by cell membrane undergoes a series of local energy minima, for which thermodynamics applies at different degrees of wrapping. We consider a single spherical NP of radius *R* being wrapped by cell membrane, as shown in Fig. 1. Wrapping partitions the membrane into three distinct regions: a small region of area 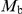 bound with the NP, an impacted region of area 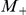 in the vicinity of the bound region, and a remote region 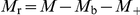 for the rest of cell membrane, where *M* is the total membrane area. We consider a general stage of wrapping characterized by the degree of wrapping 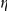. Concentrating of receptors onto the NP surface rapidly depletes the receptors in the region 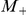 i.e., 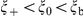, where 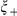 is the receptor density in the area 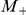. The balance of receptor potentials in the bound and impacted regions gives rise to (Methods: system free energy of single NP-membrane interaction)
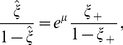
(3)where 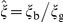. The pressure balance between the bound and impacted regions yields
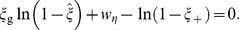
(4)At specified wrapping extent, 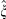 and 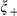 can be obtained by solving Eqs. (3) and (4), yielding the distribution of the receptors.

Since 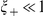, one has
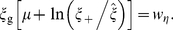
(5)Equation (5) may be interpreted as a “global” energy balance criterion for wrapping between adhesion and membrane deformation energies. In conjugation to the membrane deformation energy density 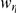,
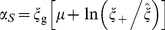
(6)is naturally defined as the adhesion strength. Here the subscript “S” stands for the case of a single NP-membrane interaction. The adhesion strength is constituted of an enthalpic (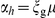) and an entropic (
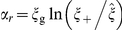
) component, representing the local and global aspects of adhesion, respectively. The entropic adhesion strength varies with the distribution of the receptors; its functional dependence on both the ligand density and particle size is indicated from Eqs. (3) and (4). Note that 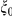 is typically on the order of 10^−2^. At 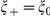, from Eq. (3) 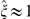, 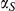 reaches its maximum 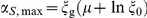, and the particle radius reaches its minimal value
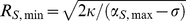
(7)for fixed 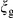.

Conservation of the receptors in the wrapping zone and the impacted area specifies a characteristic length 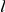 [5], [7], [16], defined by
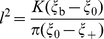
(8)The endocytic time is then *t*∼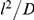, where *D* is the diffusivity of the receptors. Solving 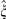 and 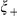 at specified 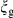 and *R*, and substituting them into Eq. (8), the characteristic impact length scale and hence the endocytic time can be obtained.

**Figure 2:**
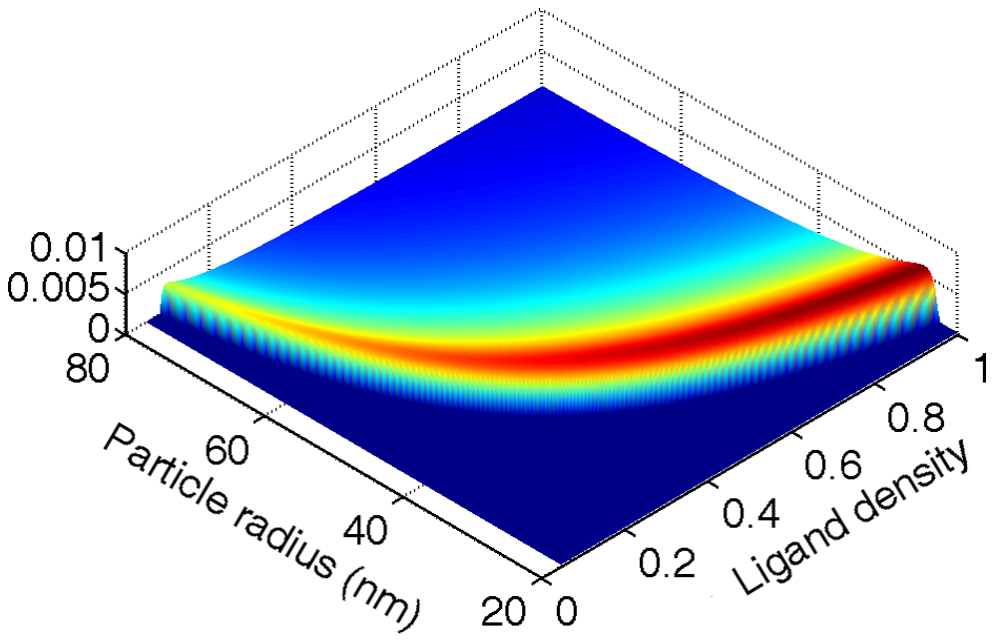
Phase diagram of 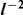 (the inverse endocytic time) in the space of particle size and ligand density.

Figure 2 displays a phase diagram of 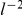
∼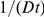 in the space of particle radius and ligand density. For particles of small size or low ligand density, wrapping consumes only a few receptors. Engulfment of the NP thus hardly changes the overall distribution of the receptors and the entropic penalty is nearly minimized, i.e., 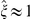 and 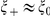, yielding a very large endocytic time (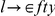). This extreme condition corresponds to the lower boundary of the phase diagram described by Eq. (8). Oppositely, at large particle sizes or ligand density, wrapping the NP consumes significant number of receptors, and 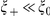. At this regime, increasing particle size or ligand density simply increases the total number of receptors (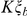) needed for wrapping, and thus the endocytic time increases. There exists a critical condition at which the receptors in the impacted region are nearly depleted (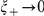); further wrapping involves significant entropic penalty. As a result, the adhesion strength becomes too low to overcome the deformation barrier. This critical condition corresponds to an upper boundary of the phase diagram at which 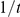 diminishes. Between these two extreme conditions, there exists an optimal condition at which the endocytic time minimizes, corresponding to the ridge line in the phase diagram in Fig. 2.

#### 3.2 Cellular uptake

**Figure 3:**
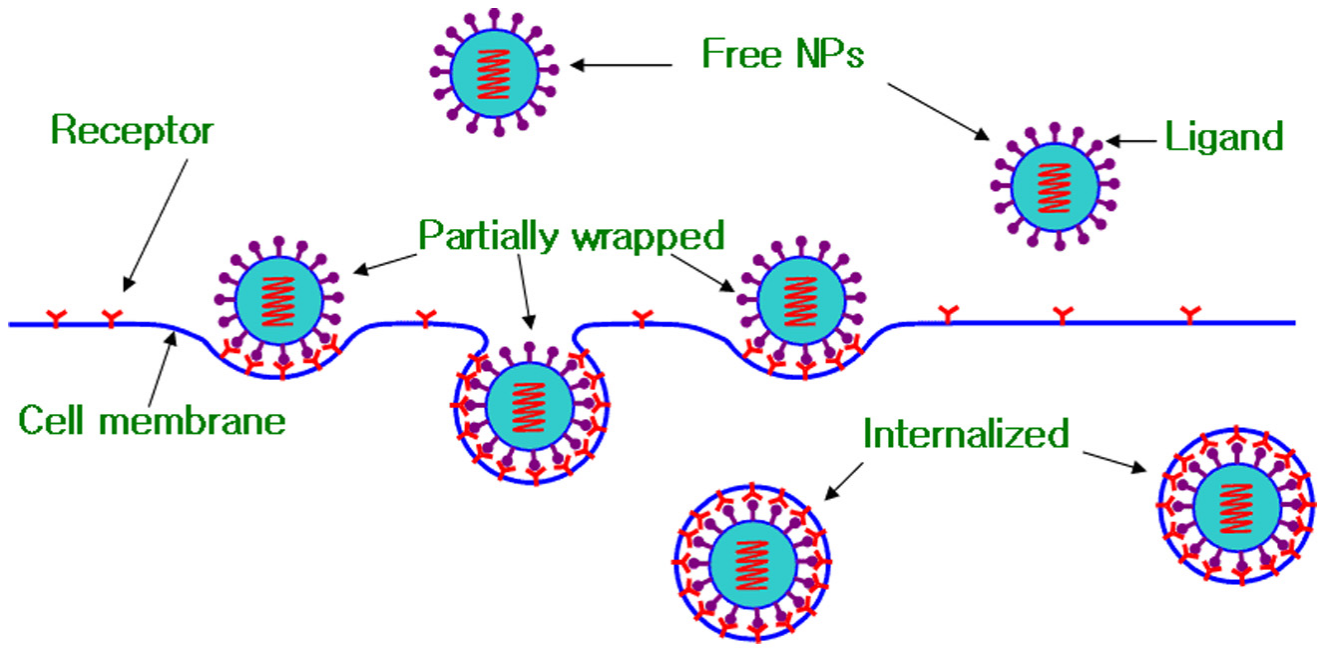
Schematics of receptor-mediated endocytosis of NPs.

We next analyze the cellular uptake of NPs when immersing the cell in a solution with dispersed NPs of bulk density 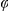. Driven by the chemical potential difference between the adherent and suspended NPs, the many-NP-cell system reaches a thermodynamic equilibrium with a steady-state cellular uptake, as suggested by a set of prior experiments [12], [13], [14]. We assume that in the thermodynamic equilibrium, a certain number of NPs, *N*, are wrapped by cell membrane with different degrees of wrapping [19], [30]; some are internalized, as shown in Fig. 3. At the thermodynamic equilibrium, the membrane is partitioned into two parts: a free, planar membrane region of area 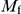 and a curved membrane region of area 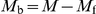 bound to the NPs. Receptors in the planar membrane region with a density 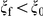 are diffusible, while those in the curved membrane region with a density of 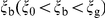 are densely packed on the NP surfaces via ligand-receptor binding. Denoting by 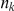 the number of NPs whose wrapped area is *k*, one follows 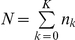 and 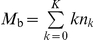 [30]. The balance of the receptor potentials in the free and bound membrane regions gives rise to (Methods: system free energy of multiple NP-membrane interaction)
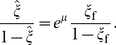
(9)One notes the close similarity of Eqs. (3) and (9). The chemical potential balance of the NPs in the bulk solution and on the cell membrane gives rise to a Boltzmann wrapping size distribution, as
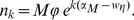
(10)where 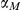 is naturally defined as the adhesion strength, where the subscript “M” stands for the case of multiple NPs interacting with the cell membrane, and
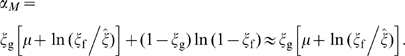
(11)The approximation in Eq. (11) holds because the last term is much smaller than the other two terms. The cellular uptake is the number of particles that are fully wrapped, i.e., 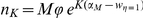. Conservation of the receptors yields:
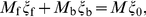
(12)Combining Eqs. (9)–(12), one finds the equilibrium densities of bound and free receptors and the wrapping-size distribution, and therefore the cellular uptake 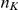.

It is noteworthy to point out the close similarities of the adhesion strengths 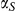 and 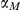 in these two cases, shown in Eqs. (6) and (11), respectively, manifesting the universal role of the adhesion strength. Taking 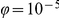
∼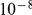, and 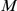
∼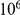, the cellular uptake nearly vanishes when 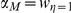. This critical condition coincides with the energy balance of adhesion and membrane deformation. In the limit of very small particle size or ligand density, wrapping hardly disturbs the receptors distribution, and 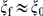 and 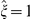. Under this extreme condition, the adhesion strength reaches its maximum, 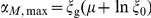. From 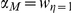, one derives a minimal particle radius,
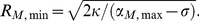
(13)Interestingly, one notes that 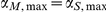, and hence 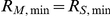.

**Figure 4:**
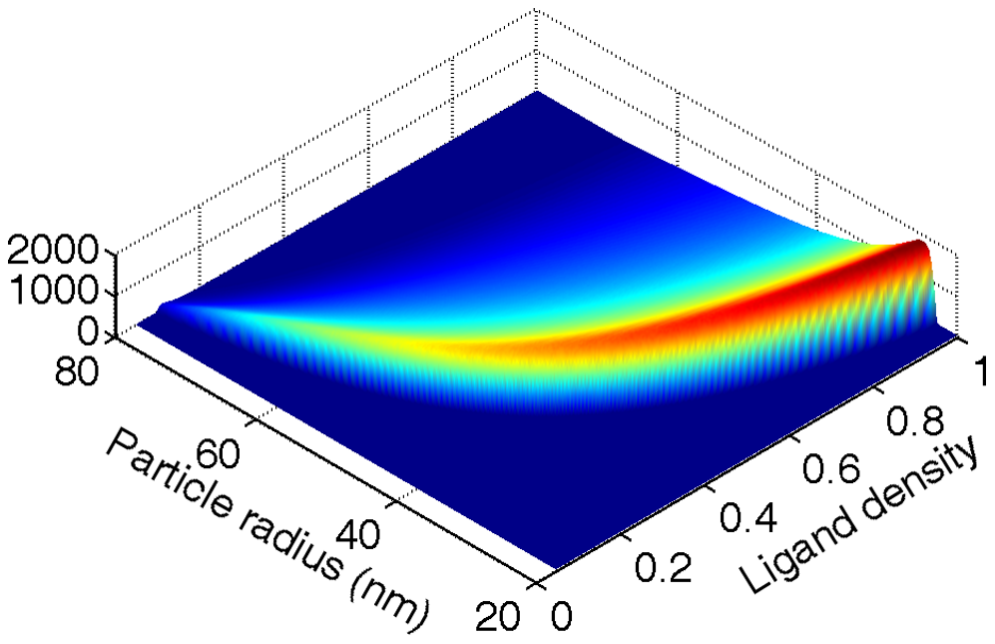
Phase diagram of cellular uptake in the space of particle size and ligand density.

Figure 4 plots the phase diagram of the cellular uptake in the space of particle radius and ligand density. This phase diagram is similar to that for the endocytic time in Fig. 2, indicating that the uptake behavior of these two cases shares the same mechanism. Based on Eqs. (7) and (13), the lower bounds of these two phase diagram are exactly the same. As the particle size and ligand density increase, increasingly more receptors diffuse toward the wrapping sites, and the receptors in the free membrane regions are nearly depleted, i.e., 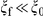. Under this condition, the entropic penalty becomes significant and substantially lowers the adhesion strength. Corresponding to this entropic limit, an upper bound exists at which the cellular uptake vanishes.

#### 3.3 Uptake rate

The unified energy-balance framework of adhesion and membrane deformation for the endocytic time and the cellular uptake suggests that one may define the uptake rate as
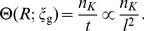
(14)Since both 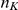 and 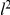 depend on particle size and ligand density, so as the uptake rate 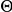.

**Figure 5:**
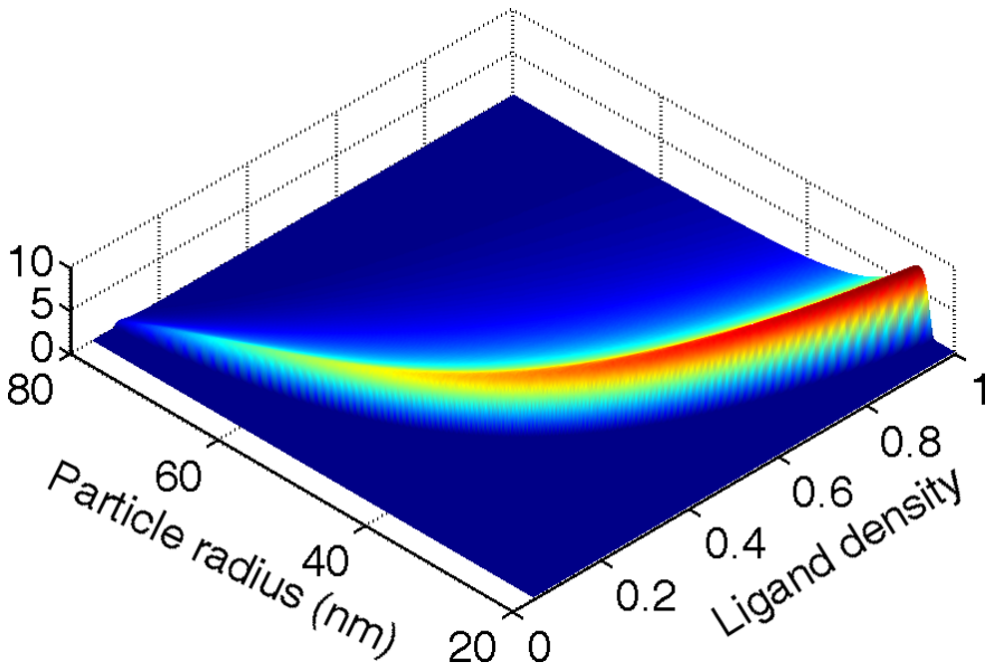
The phase diagram of uptake rate in the space of particle radius and ligand density.

Figure 5 plots the phase diagram of the uptake rate in the space of particle size and ligand density. Similar to the phase diagrams for the endocytic time and cellular uptake, there exists a lower and an upper bound for the phase diagram at which 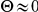. According to Eq. (14), the lower and upper bounds can be reached at limiting conditions 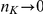 or 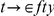 or both. Our previous analysis showed that at the same lower bounds, represented by Eqs. (7) and (13), 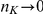 and 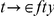 are simultaneously reached. The lower bound corresponds to the enthalpic limit of the adhesion strength. The upper bounds at which 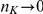 and 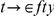 arrive are seemly quantitatively different. However, the upper bounds are governed by the same mechanism, i.e., they are set by the entropic limit of the adhesion strength. Due to the competition for receptors among NPs in the case of multiple NP-cell interactions, the entropic limit of the cellular uptake is much easier to reach as compared to that of the endocytic time. One thus follows that the upper bound of the phase diagram for the uptake rate is due to the vanishing cellular uptake (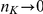).

From the phase diagram of the uptake rate in Fig. 5, we identified a small region at which the uptake rate reaches the global maximum. This region corresponds 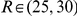 and 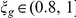. The optimal range of particle size coincides with the experimental data [10], [13], [14], and is also consistent with the typical size of virus. The overall optimal range of ligand density indicates that the maximal uptake rate is achieved when nearly every ligand binds with a receptor. Previous analysis showed that the density of bound receptors in virus budding is nearly saturated [30], which indirectly supports our results.

**Figure 6:**
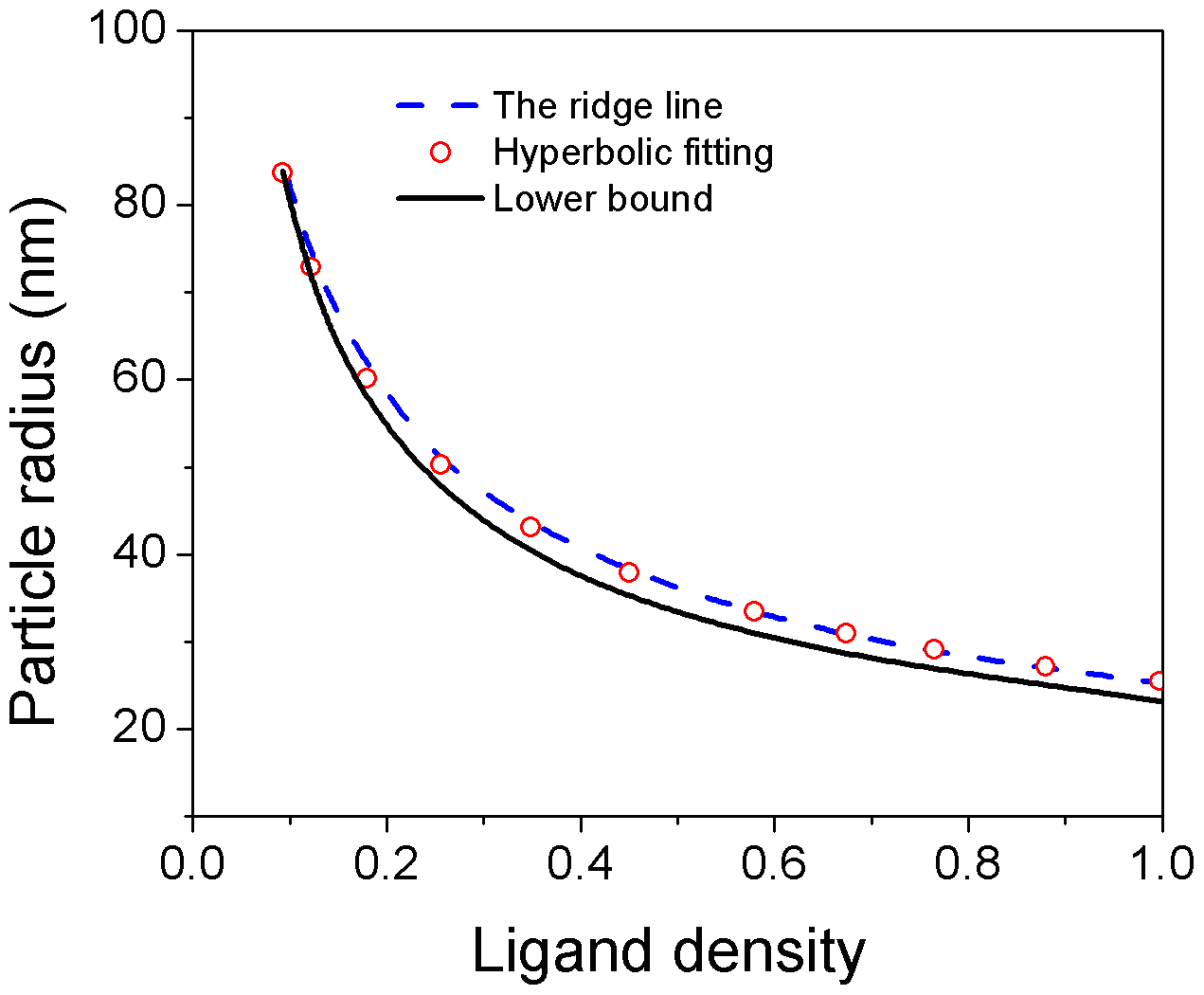
The lower bound and the ridge line extracted from the phase diagram of the uptake rate. The ridge line (optimal condition) follows a hyperbolic fitting (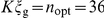).

The ridge line of the phase diagram in Fig. 5 represents the optimal condition at varying particle size. Figure 6 plots the lower bound and the ridge line extracted from Fig. 5. Recall that the lower bound can be written as 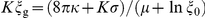. Neglecting the membrane tension effect, the lower bound can be approximated 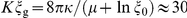 (for 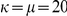 and 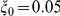). Figure 6 shows that the ridge line is fairly close to the lower bound, suggesting that the optimal condition can be approximated by 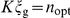, where 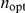 is the optimal number of ligands that should be coated onto the NP surface. Indeed, we found that the ridge line follows well a hyperbolic fitting 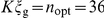. It should be pointed out that this optimal number is independent of the particle size.

Considering viruses as NPs optimized by nature via evolution, the number of ligands decorated on the surfaces of viruses should obey the optimal number: 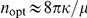. However, as discussed later, in physiological conditions, both the bending rigidity and the receptor-binding energy are subjected to change to certain extents. Given the biophysically relevant ranges for membrane bending rigidity (10–40 *k*_B_ *T*) and receptor-ligand binding energy 
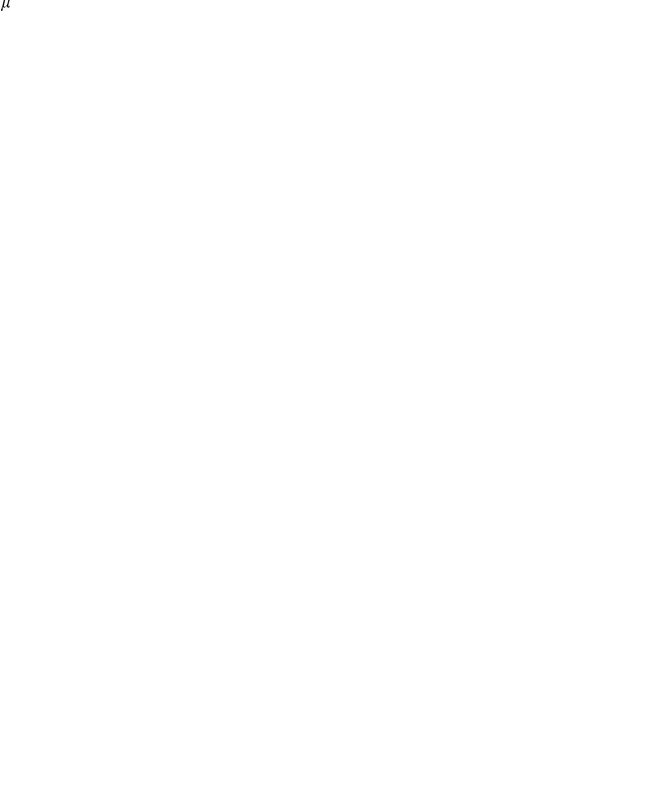
 (10–20 *k*_B_ *T*), our model predicts that the optimal amount of ligands coated onto viruses falls in the range 10–100 irrespective of the virus size. The extensively studied model system, the Semliki Forest virus (SFV), is about 35nm in radius, covered with 80 glycoproteins (ligands) [32], [33]. The SFV structures seem to agree with our model predictions.

### 4. Effects of other biophysical parameters

In addition to the particle size and ligand density, many factors may influence the phase boundaries of the uptake rate, as presented below.

#### 4.1 Effects of 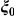


**Figure 7:**
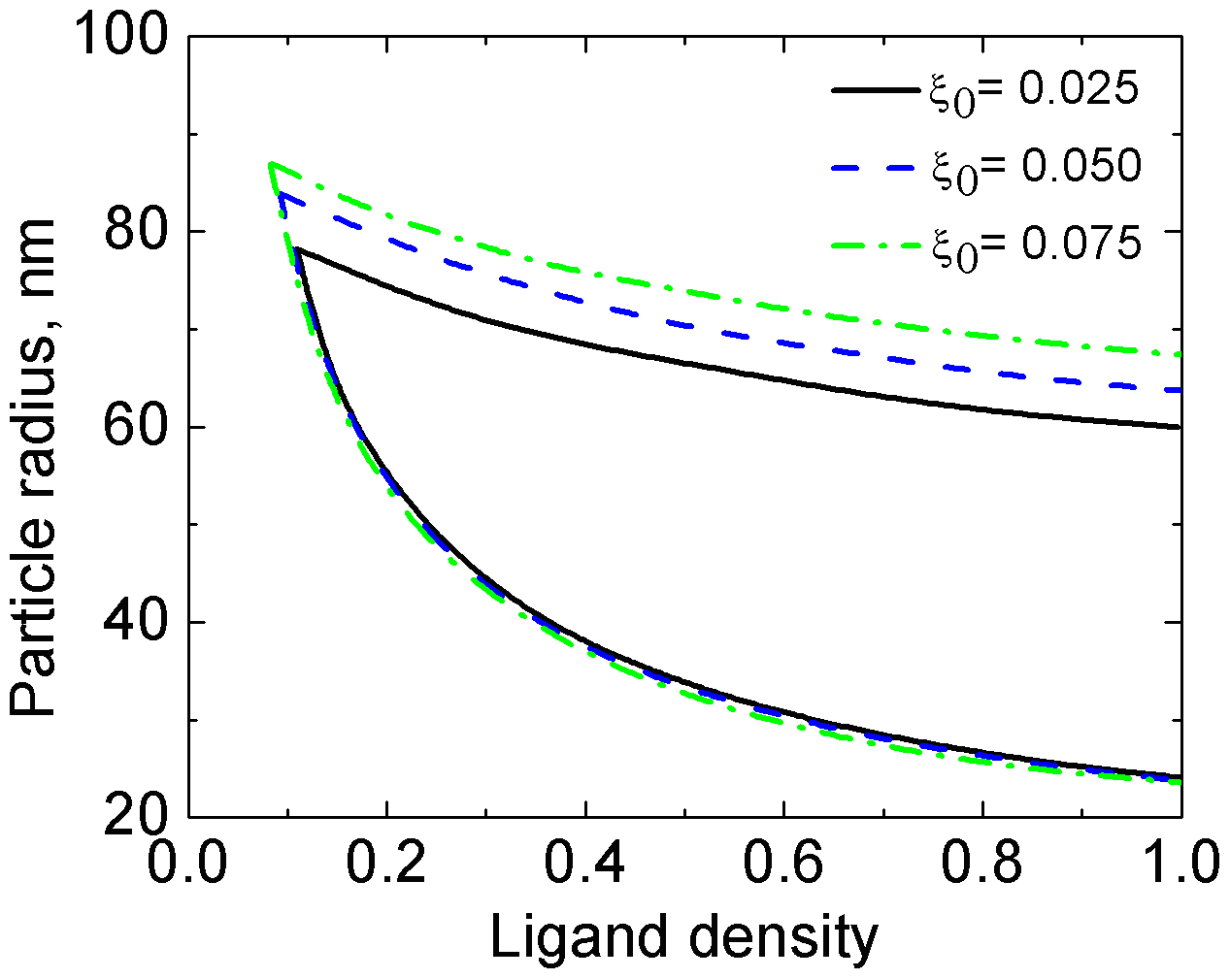
Increasing the receptor density 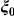 shifts the upper bound of the phase diagram upward, but hardly affects the lower bound.

Several factors affect the density of receptors expressing on the cell membrane. First of all, receptors internalized by NPs may be recycled back to the host membrane; they may also be degraded in the endosomes and lysosomes. In addition, new receptors may be produced and diffuse to the cell membrane. The precise amount of receptors involved in endocytosis is currently unknown. Figure 7 plots the lower and upper bounds of the uptake rate at three different values of 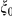. Due to the weak dependence of the lower bounds of the endocytic time (Eq. (7)) and of the cellular uptake (Eq. (14)) on 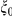, the lower bounds of the uptake rate are nearly the same at the three values of 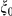. With increasing 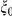, the increasing population of receptors lowers the entropic penalty for the receptors to bind with NPs. This effectively increases the adhesion strength. Since the upper bound of the uptake rate is entropically dominant, it shifts upward with increasing 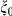, as shown in Fig. 7.

#### 4.2 Effect of relative energy scale

**Figure 8:**
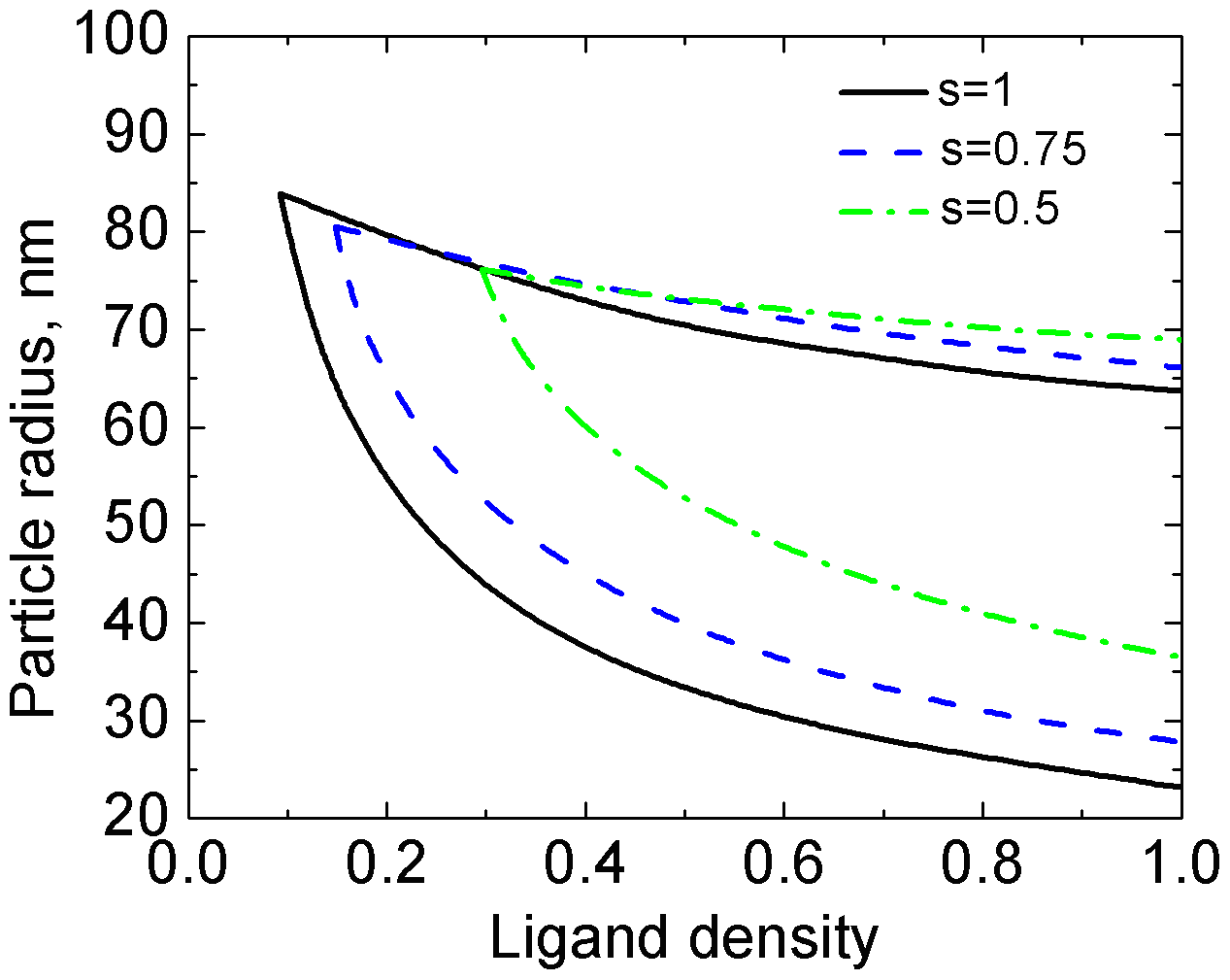
The relative energy scale *s* regulates the lower bound of the uptake rate, but only weakly affects the upper bound.

In addition to ligand-receptor binding, receptor-mediated endocytosis may be assisted by specific proteins, such as clathrin or caveolin [34], [35], [36], contributing to additional driving force to locally curve the membrane. Nonspecific interactions [24], such as hydrophobic forces, electrostatic forces, and van der Waals interactions, may also contribute to additional adhesion energy. Lumping the specific and nonspecific interaction together, one can determine an effective ligand-receptor binding energy, 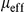. Bending rigidity represents the energy scale that resists wrapping, which may also vary for different cell types. When the cortical actin network plays a role in endocytosis [37], the effective bending rigidity 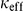 increases. We define the relative energy scale by 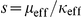, with which we construct the phase diagram of the uptake rate, as shown in Fig. 8.

One notes that variation of the relative energy scale leads to the change of the enthalpic component of the adhesion strength and/or the membrane deformation energy density. As the lower bound of the uptake rate is enthalpically governed, variation of the relative energy scale modifies the lower bound of the phase diagram. On the other hand, the upper bound is entropically governed, and thus only weakly dependent on 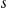, as shown in Fig. 8. The weak dependence arises from the change of the membrane deformation energy density relative to the adhesion energy density.

#### 4.3 Effects of membrane tension and NP bulk density

**Figure 9:**
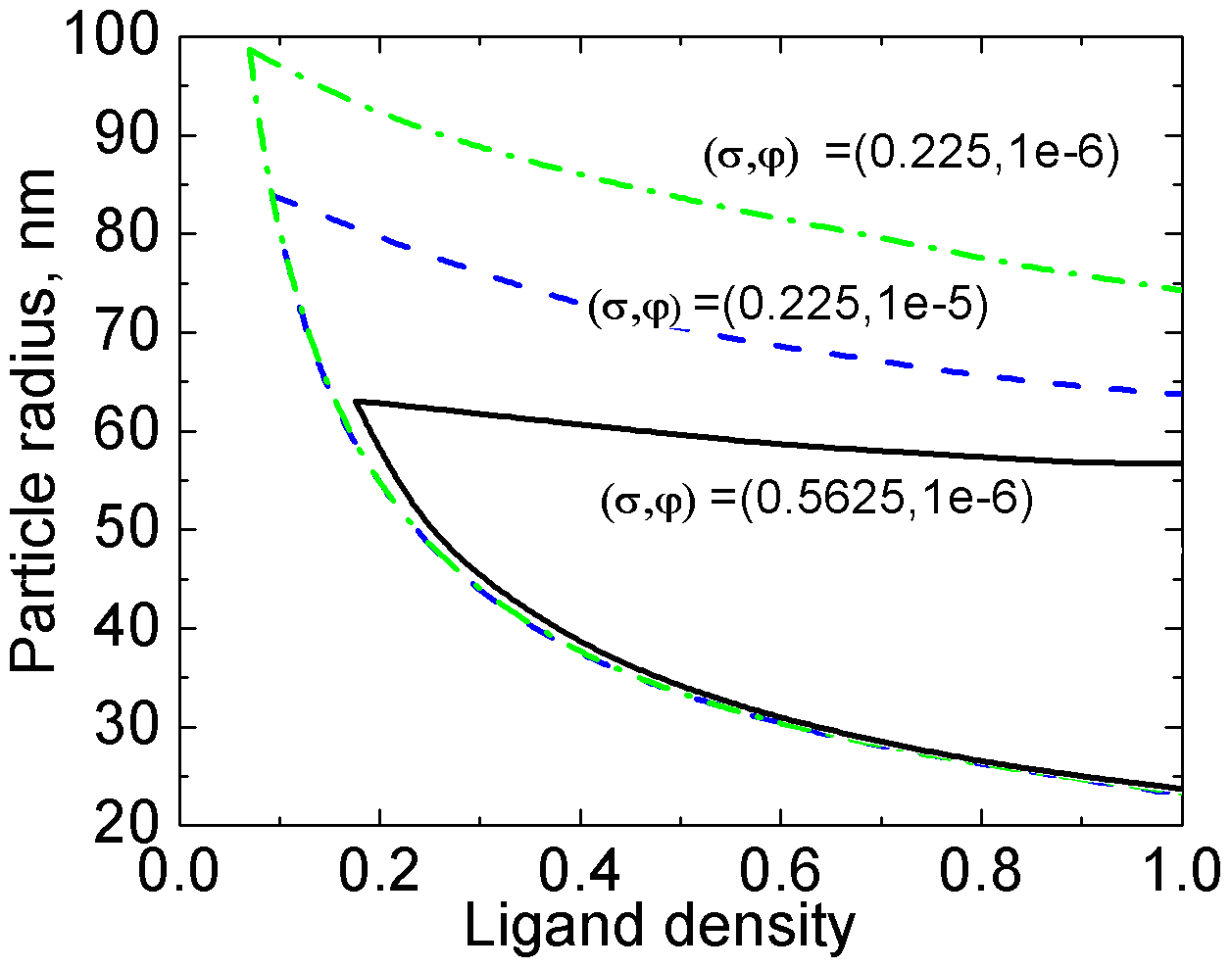
Membrane tension and the bulk density of NPs regulate the upper bound of the uptake rate, but hardly affect the lower bound.

The bulk density of NPs in solution appeared as a model parameter only for computing the cellular uptake. A high bulk density yields a high surface concentration of NPs on cell membrane, leading to intensified competition for receptors among adhering NPs [20], [31] and high entropic penalty for concentrating receptors onto NP surfaces. This follows that increasing the bulk density of NPs decreases the adhesion strength, and therefore shifts the upper bound of the uptake rate downward. Since the bulk density only affects the entropy of the receptors, the lower bound of uptake rate is hardly affected, as shown in Fig. 9. Cells may actively modulate their membrane tension under different physiological conditions through various mechanisms such as membrane reservoir release, lipid molecules insertion into cell membrane, interference from the cortical actin network [37], etc. A high membrane tension corresponds to a high deformation energy cost, and hence increases the endocytic time and reduces the cellular uptake and uptake rate. We have pointed out that the relative significance of the membrane tension and bending defines a characteristic particle radius 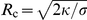 beyond (below) which membrane tension (bending) dominates the physics. Thus, the effect of membrane tension is negligible for small particles but significant for large particles. This follows that membrane tension primarily regulates the upper bound of the uptake rate, but hardly affects the lower bound, as shown in Fig. 9.

## Discussion

Through thermodynamic analyses, we revealed that the endocytic time of a single NP and the cellular uptake when immersing the cell into a solution with dispersed NPs are governed by the unified framework of energy balance between adhesion and membrane deformation. We established phase diagrams in the space of particle size and ligand density for both the endocytic time and the cellular uptake. We identified from the phase diagrams the lower (upper) bounds below (beyond) which the endocytic time goes to infinite or the cellular uptake vanishes. We further revealed that the mechanisms governing the lower and upper bounds of the endocytic time and the cellular uptake are the same: the lower bounds correspond to the enthalpic limit of the NP-membrane adhesion strength, while the upper bounds to the entropic limit.

The computed endocytic time and the cellular uptake allow us to define the uptake rate. It should be mentioned that the uptake rate defined here is different from what is typically measured in experiments [13] since the complex dynamics of receptor binding and debonding with NPs is not fully taken into account. However, it may still serve as an important index to assess the uptake efficacy of NP-based therapeutics. The optimal size at which the uptake rate maximizes agrees with experimental data [8], [13], [14], [18]. Our model also predicts that, optimally several tens of ligands should be coated onto the NP surface in order to achieve high uptake rate. These findings are supported both by the experimental data and the typical viral structures. The interrelated dependence of the uptake rate on the particle size and ligand density predicted by our analysis invites well-controlled experiments for further validation.

We further discussed the effects of other relevant biophysical parameters on the uptake rate, including the receptor density, the relative energy scale of ligand-receptor binding energy and membrane bending rigidity, membrane tension, and the bulk density of NPs. All the effects can be coherently interpreted by the variation of the enthalpic and entropic adhesion strength. The phase diagram of the uptake rate in the space of particle size and ligand density thus serves as a design map that guides the rational designs of NP-based bioagents for biosensing [38], [39], bioimaging [40], [41], and drug delivery [42], [43].

## Methods

### 1. System free energy of a single NP-membrane interaction

We consider a general stage of wrapping at which an area of 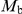 is wrapped by 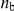 receptors. By definition, the receptor density in the wrapping zone is 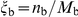. The impacted region of area 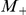 in the immediate vicinity of the wrapping zone with an average receptor density is 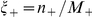, where 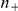 is the number of receptors in the region of area 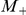. The free energy in the area 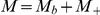 can be written as:
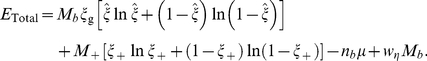
(A1)The first two terms in Eq. (A1) are the translational entropy of the receptors in the bound and free membrane regions, and the other two terms are adhesion and bending energies, respectively. Considering the constraints of conservation of membrane area 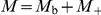 and conservation of receptors 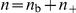, the free energy functional features two independent variables: 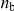 and 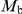. Minimizing the energy functional with respect to these two independent variables subject to these constraints gives rise to the equilibrium conditions Eqs. (3) and (4). It should be noted that in the case of 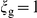 our free energy functional derives the equilibrium conditions in the analysis of Bao and Bao [5].

### 2. System free energy of multiple NP-membrane interaction

Corresponding to the wrapping-size distribution 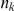, a total free energy functional for the system takes the following form:
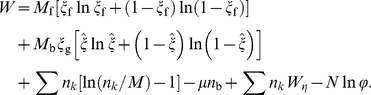
(A2)The first three terms are entropic contributions: the first two terms are the translational entropies of the bound and free receptors, respectively; the third term accounts for the configurational entropy of the 2D mixture of wrapped NPs, treated here as a multi-component ideal gas. The next three terms are energetic contributions: 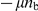 is the chemical energy release upon the binding of 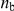 ligand-receptor pairs. The second energetic term lumps over the total deformation energy of the membrane. The last term is the energy penalty involved in NP adsorption to the membrane [17].

The thermodynamic equilibrium, expressed by Eqs. (10) and (11), can be obtained by minimizing the free energy functional with respect to its two independent variables, 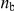 and 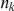. It has been argued [17] that for a more general consideration the wrapping size distribution should follow a two-dimensional distribution 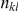, which represents the number of NPs wrapped by an area *k* using *l* receptors. In the present analysis, we assumed that the density of receptors bound to NPs is independent of wrapping size. This simplification does not affect the qualitative conclusions drawn here.
